# Molecular Perception for Visualization and Computation: The Proxima Library

**DOI:** 10.1021/acs.jcim.0c00076

**Published:** 2020-04-09

**Authors:** Federico Lazzari, Andrea Salvadori, Giordano Mancini, Vincenzo Barone

**Affiliations:** Scuola Normale Superiore, Piazza dei Cavalieri, 7−56126 Pisa, Italy

## Abstract

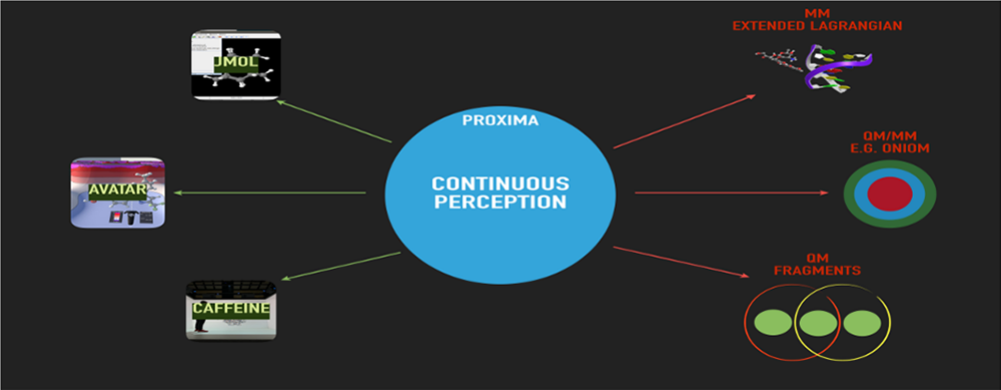

Proxima is a molecular perception library designed with a double purpose: to be used with immersive molecular viewers (thus providing any required feature not supported by third party libraries) and to be integrated in workflow managers thus providing the functionalities needed for the first steps of molecular modeling studies. It thus stands at the boundary between visualization and computation. The purpose of the present article is to provide a general introduction to the first release of Proxima, describe its most significant features, and highlight its performance by means of some case studies. The current version of Proxima is available for evaluation purposes at https://bitbucket.org/sns-smartlab/proxima/src/master/.

## Introduction

The goal of molecular perception algorithms is to build topological models of molecular systems starting from the minimal information conveyed by atomic symbols and coordinates, to be next used for scientific visualization purposes, or for building initial guesses for quantum mechanics (QM) and/or molecular mechanics (MM) simulations. As a matter of fact, molecular perception has been (quite restrictively) defined as “the process of distinguishing between different chemical environments in order to assign force field parameters”.^1^

**Figure 1:**
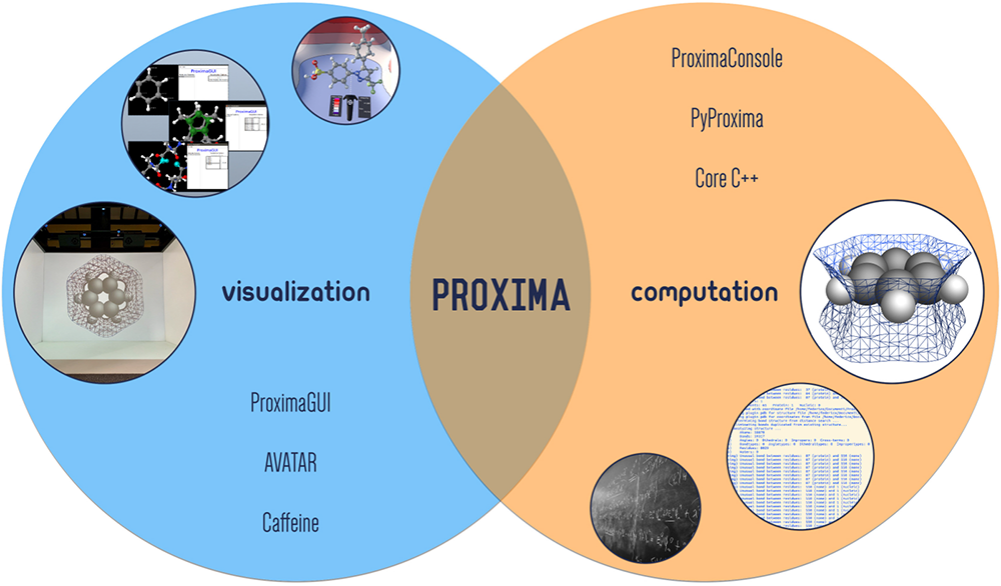
Proxima is a molecular perception library aimed at computational chemistry but also employable by molecular visualization software.

In this work, we present Proxima, the molecular perception library under development at the SMART laboratory of Scuola Normale Superiore.^2^ The purpose of Proxima is (i) to provide information about bonds, rings, atom types, and related data to imersive visualization software (e.g., Caffeine^3^), (ii) to compute molecular properties such as atomic charges, atomic hybridization, or hydrogen bonds, (iii) to be connected to workflow managers in order to exploit property calculations to prepare input files, and (iv) to allow user-friendly use also by nonexperts. In other words, our aim is to build a tool standing in the middle between computation and visualization (see Figure 1).

We tried to explore some innovative strategies for molecular perception like, e.g., the computation of continuous bond orders by setting sensible limits to atomic valence and thus taking into account uncertainty in the starting atom coordinates.

As an example of application of Proxima for computational purposes, the unsupervised computation of biaryl torsional parameters in complex systems is analyzed. For visualization tasks we demonstrate the remarkable flexibility of the library by showing its use in conjunction with Caffeine, the stand alone ProximaGUI, and the AVATAR Virtual Reality application^4^ for exploring potential energy surfaces computed by Proxima itself.

## Methodology

### Perception

Starting from Cartesian coordinates and atom numbers (see the section about the Proxima I/O in the Supporting Information) we proceed with chemical perception. The first step is the perception of different kinds of bonds (currently, covalent and hydrogen bonds are perceived).

#### Bond Perception

Several characteristics (e.g., strength, polarity, etc.) of chemical bonds are tuned by the nature of the involved atoms and by their environment. We decided to use the same mathematical form not only for detecting the presence of a bond, but also for evaluating its strength. In other words, Proxima is based on the concept of continuous chemical perception. In particular, the strength of a bond can be written as
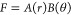
1Where *B*(θ) is an angular term required for three-body interactions (e.g., hydrogen bonds). Both the A and the B functions have the same mathematical form shown below for the radial component:
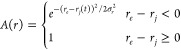
2This function is continuous also with its first derivative. The nature of the parameters (*r*_*e*_ and σ_*r*_ in the example) depends on the type of bond considered.

#### Covalent Bonds

Traditionally, the computation of the covalent bond connectivity is performed by evaluating the distance between each pair of atoms without further corrections related to atom types. If the distance between two atoms is longer than a small threshold (here 0.4 Å) and shorter than the sum of their respective covalent radii, plus some “tolerance” factor (usually 0.4 Å^7^), then a covalent bond is assigned to the given pair. We increased the sensitivity of this formulation by including the electronegativity in the theoretical bond length expression, in a way that has been proposed for the first time by Schomacker and Stevenson^8^ and then improved by Porterfield:^9^
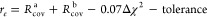
3This is the lower bound to the single covalent bond length (the tolerance is taken as 0.4).

Here, *r*_*e*_ is the distance parameter used in eq 2 (in Angstroms), *R*_cov_^a^ and *R*_cov_^b^ are the covalent radii for the atoms “a” and “b” respectively, *Δχ* is the electronegativity difference between the two species (values are taken from a work of Little and Jones^10^), whose contribution is significant especially when dealing with ionic interactions. Since the covalent bond is a two-body interaction, only the radial component of eq 1 is considered, and the σ_*r*_ of eq 2 is taken as
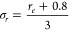
4

**Figure 2:**
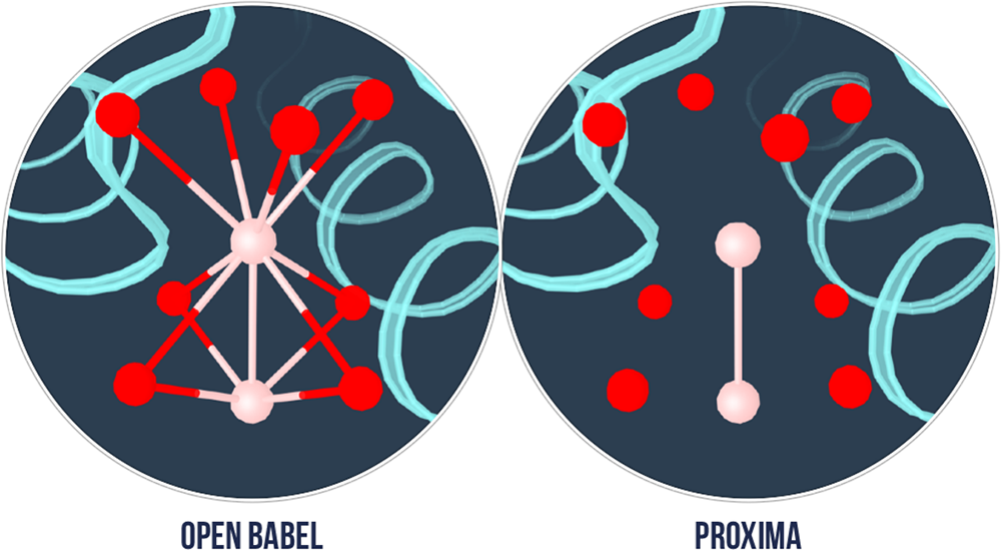
Looking at a difficult case: the potassium atoms within the potassium channel (PDB ID: 1BL8). (left) Bond perception performed by the Avogadro software^5^ (version 1.1.1) (using the OpenBabel library^6^ (version 2.3.2)): number of bonds perceived, 2918. (right) Bond perception performed by Proxima: number of bonds perceived, 2897.

A new covalent bond is assigned only if the strength of the new bond is above 0.4. An example is the potassium channel of Figure 2. Here, OpenBabel 2.3.2 overestimates the number of covalent bonds with the potassium atoms, whereas Proxima correctly describes such system thanks to a proper account of the electronegativity contribution (see eq 3). Proxima also checks the consistence of bond assignments by removing excess bonds that make the coordination of the atom exceed its valence. Once bonds are detected, valence and dihedral angles can be straightforwardly determined.

#### Hydrogen Bonds

Following a simple geometric approach widely used in MM, if the donor–hydrogen–acceptor geometry is compatible with a reference geometry, then an hydrogen bond is assigned to such an assembly of atoms. Since this is a three-body interaction, we use the general expression of eq 1 including also the angular contribution (θ is the donor–H–acceptor angle).

Proxima employs the parameters proposed by Pagliai^11−13^ and supports nitrogen and oxygen atoms both as donors and acceptors.

#### Ring Perception

Proxima implements the Horton’s algorithm for the perception of chemical rings.^14^ Despite its worst-case complexity of *O*(*n*^7^), the algorithm can be applied only to the cyclic portions of the system (called blocks in the following), which represent a very small part of the molecular systems of current interest for Proxima applications (e.g., proteins). To this end, we have implemented the block partitioning scheme suggested by Fan, Doucet, and Barbu.^15^ Some benchmarks are shown in the Supporting Information.

#### Hybridization Perception

The perception of hybridization is performed by looking at the bond angles around each atom. This is similar to the OpenBabel approach, with some additional tweaks like, e.g. the hybridization of the most common transition elements. After computing the minimum and the maximum bond angles for each atom, Proxima assigns the hybridization in relation to an heuristic, provided that the considered atom has a sufficient number of bonds. Thus, terminal atoms must be processed separately. Moreover, it is important to note that in very small ring structures the values of the angles are not really representative of the hybridization state. Thus, there are two categories of atoms that Proxima processes separately: (i) cyclic planar atoms and (ii) terminal atoms. The hybridization of all atoms belonging to planar cycles is taken as an sp^2^. Next, the average torsion angle of those atoms is computed. Typically, the hybridization of terminal atoms is left unknown. There are some special cases, though, that are explicitly treated such as oxygen, nitrogen, and carbon atoms. For cycles that have four or five bonds, the cycle is considered to be planar only if the mean torsion angle is below 7.5°, whereas this threshold is increased to 15° for larger cycles. Further details are included in the Supporting Information.

#### Charges and Bond Orders

Electrostatic interactions are the longest ranged contributions in MM force fields and usually account for the largest fraction of the total energy. Therefore, their computation is a critical component of any FF and the simplest and most widespread approach is the computation of fixed point charges assigned to atoms. These can be either perceived directly on the basis of tabulated quantities (e.g., electronegativity) and coordinates or be the results of a quantum chemistry calculation (e.g., RESP^16^ or Hirshfeld^17^ charges). The possibility of computing charges with a cheap perception model can be a useful feature in the initial stages of a study, so that several perception libraries and molecular editors offer such feature. Along these lines Proxima allows computation of the point charges using the Gasteiger^18^ (popular in the medicinal chemistry domain) and the QeQ^19,20^ (which is the default method of assigning charges in UFF) methods. Both methods depend on tabulated atomic parameters and possibly on more refined atom types (the CHARMM ones^21^ for QeQ).

Concerning bond orders, the goal of Proxima is to compute continuous quantities instead of the discrete ones usually employed by other software. We have already implemented a method based on bond lengths, which is, however quite error prone for structures with low spatial resolution. As a consequence, we have also developed a custom filtering procedure, which enforces correct boundaries. It is important to notice how we decoupled the problem of finding bond orders from the problem of finding the strength of a bond.

#### Distance Based Bond Order

The custom Proxima method for computing bond orders is based on the knowledge of the covalent radii for single, double and triple bonds for all the elements of the periodic table, which were tabulated by Pyykkö.^22^

In order to move from a discrete to a continuous approach, we have fitted the three covalent bond radii of each element (*r*_cov,*s*_, *r*_cov,*d*_, and *r*_cov,*t*_) with the corresponding bond order values (1, 2, and 3). The function used to fit such quantities is an exponential:
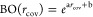
5Here, *r*_cov_ is the analogous of the covalent bond radius in a continuous formulation and represents the contribution of the atom to the total bond length. BO is the overall bond order for the bond. By simple algebraic manipulations, one obtains:
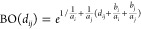
6

#### Proxima Filtering Procedure

As mentioned above, distance-based bond orders show a huge dependence on the reference molecular geometry. However, it is possible to check the consistency of a given bond order assignment by looking at the sum of bond orders around each atom. If this value is not compatible with the valence of the atom itself, then the given bond orders are not the correct ones. Minimization of the squared distance between the new bond orders and the original values with the constraint of correct atom valences leads to a Lagrange multiplier problem, which is solved in Proxima by standard routines of the Eigen linear algebra library.^23^

### Application to Biaryl Systems

As a case study, we consider the problem of finding torsional parameters for biaryl groups in molecules. A recent work by Liu, Barigye, Shahamat, Labute, and Moitesser^24^ has shown that it is possible to relate one torsional parameter to the difference in electronegativity between the atoms of the two aromatic rings involved in the biaryl. The software Liu^24^ developed (called HTEQ) is a Java application that takes a mol2 file containing the biaryl molecule and a text file containing the serial numbers of the atoms of the inter-ring bond as inputs. The goal was to use the perception algorithms of Proxima for

- Finding bridge bonds
- Extracting the local geometry of the biaryl group from the overall system

#### Finding Bridge Bonds

In order to find bridge bonds, Proxima iterates over each couple of planar rings found in the system, tagging as bridge bond any bond connecting one atom of the first ring with one atom of the second ring. Next, the serial numbers of the atoms involved in each bridge bond are stored in the format required by the HTEQ software.

#### Extracting Local Geometry

Once bridge bonds have been found, Proxima starts “cutting” the molecular system around each of them in order to isolate the biaryl group from the rest of the molecule. This system is then stored in the mol2 file required as input by the software that computes the torsional parameters. The combination of this cutting system with the serial numbers of the bridge atoms (retrieved in the previous step) provides all the information needed by the HTEQ software.

**Figure 3:**
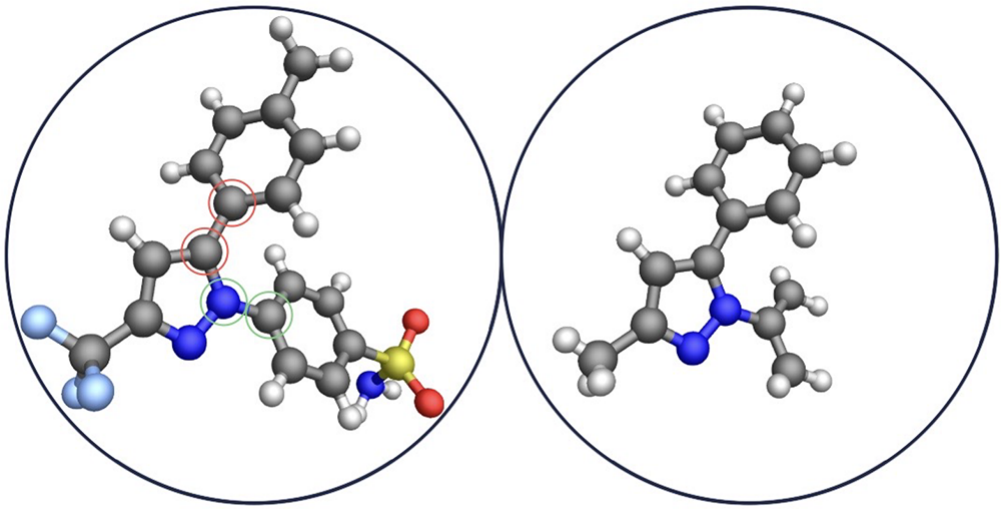
Celecoxib molecule on the left. On the right, the molecular system cut at a distance of four bonds around the red atoms.

As an example, in Figure 3 a celecoxib molecule is shown. Here, the highlighted atoms are the bridge atoms of this system automatically detected by Proxima. Selecting a cutting distance of four bonds, one obtains the “cut” system around the red bridge shown on the right side of Figure 3, where the cut atoms are replaced by hydrogen atoms placed at the correct distance (that is the sum of the respective covalent radii). In real applications, higher cutting distances are recommended. In this specific case, the HTEQ software predicts a V2 torsional parameter of −1.142 kcal/mol for the bridge bond highlighted in red, and a V2 torsional parameter of −0.942 kcal/mol for the bridge bond highlighted in green.

### Immersive Virtual Reality (IVR) Viewers

Proxima can be used as perception tool by our in-house IVR molecular viewer Caffeine.^3^ Moreover, the computation of molecular electrostatic potential surfaces is currently implemented in Proxima. This method uses the atomic charges (either Gasteiger or QeQ charges) to compute the electrostatic potential in each voxel of the bounding box (whose dimensions are chosen by the user) for the given molecular system. These electrostatic potential data can be saved in a cube file,^25^ which can be visualized by several visualization tools such as VMD,^26^ JMol,^27^ etc.

#### Computation and Virtual Exploration of Potential Energy Surfaces

To show the versatility of Proxima, we present a test case for the computation of potential energy surfaces with Proxima and their visualization in interactive VR environments. In particular, we are working on a virtual reality application (called AVATAR^4^), developed using the popular Unity game engine,^28^ for the interactive visualization of three-dimensional energy surfaces with the HTC Vive VR headset.^29^ The interesting aspect of such application is not only the visualization of the energy surface in virtual reality but also the possibility for the user to walk onto the surface and see how the molecular geometry changes along the path. For this test case we used Proxima to compute the potential energy surface (PES) of the celecoxib molecule associated with the rotation of the two inter-ring torsion angles (see Figure 3), while keeping the other coordinates frozen.

**Figure 4:**
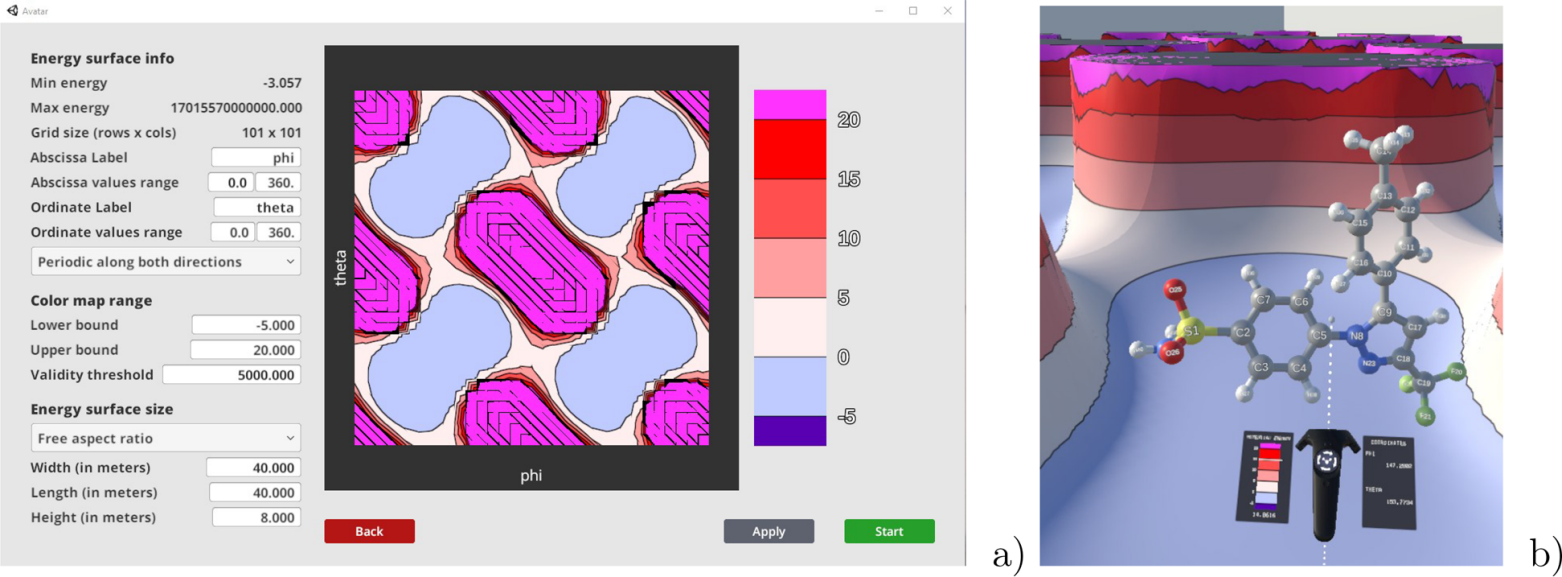
(a) Screenshot of the configuration panel of the AVATAR application^4^ for the exploration of the potential energy surfaces. The computation of the shown PES has been performed by taking into account the van der Waals, electrostatic, and V2 torsional components of the energy and relies on Proxima for the computation of the V2 parameters, the charges, the input, and the output of files. (b) Celecoxib molecule on its potential energy surface.

We have employed the Python version of Proxima (PyProxima) to rotate the molecule along the torsion angles (from 0° to 360°), to compute the V2 torsional parameter, the charges and the bond connectivity of the molecule and to output the results in a custom version of the multimodel xyz file format compatible with the VR application. The calculation of the van der Waals energy for each conformation has been performed in an external Python script, based on PyProxima, by employing the original parameters of Rappe, Casewit, Colwell, Goddard, and Skiff.^30^Figure 4a and b shows the resulting energy surface and two screenshots of the AVATAR application.^4^ As can be noted, the molecular conformation associated with the underlying point of the surface is displayed on the right controller with the corresponding energy value and updated in real time. In particular, the surface presents some divergent points where the energy rises drastically due to the overlapping of two hydrogen atoms of the two different rings (e.g., where both the torsions are equal to 0°). Moreover, the point with the lowest energy shows a geometry compatible with the one resulting from a UFF geometry optimization.

The examples described in the preceding sections show how Proxima can be exploited in tandem with other software for the interactive visualization of molecular systems and related properties.

## Conclusions and Perspectives

In this paper, we have introduced a new molecular perception library called Proxima. This library is provided with parsers for pdb, xyz, and mol (v3000) file formats. Concerning molecular perception, Proxima can detect or compute the covalent bond connectivity, chemical rings within molecular systems, atomic hybridizations, and atomic charges (Gasteiger or QeQ).

It is worth noting that, in analogy with many other available perception methods, the information detected or computed by Proxima is sufficient to perform atom type assignment; in fact, this is what many packages do, linking the perception to a domain (e.g., Antechamber with AMBER^31^ or GAFF^32^) or general (e.g., UFF^33^) FF.

From the visualization point of view most of the outputs of Proxima can be visualized in Caffeine.^3^ In addition to the Proxima C++ core library, we have also developed additional tools, allowing a greater flexibility and ease of use: PyProxima, a Python module that enables the use of Proxima in scripts or in the python interpreter, and ProximaConsole, a console application for using Proxima in the direct computation of the desired molecular properties.

Finally, we have analyzed in some detail a test case in which PyProxima has been exploited for the computation of a potential energy surface for the conformational analysis of the Celecoxib molecule, to be provided as input to the AVATAR^4^ VR application.

## Supporting Information Available

Further details about the parsing of PDB files highlighting the way residues and alternate locations are treated. Moreover, some benchmarks for the computation of the Horton’s rings are reported. Also, the complete set of rules used for the computation of the hybridization of the atoms is shown. Lastly, Caffeine, PyProxima, ProximaConsole, and ProximaGUI are discussed more in detail by also showing some pictures of the software (PDF)
