# Supplementary material for: Molecular Perception for Visualization and Computation: The Proxima Library
Source: J Chem Inf Model. 2020 Apr 9;60(6):2668–72. doi: 10.1021/acs.jcim.0c00076 (PMC7997373; doi:10.1021/acs.jcim.0c00076)
Supplement: Supplementary file 1 — ci0c00076_si_001.pdf [file ci0c00076_si_001.pdf]

Molecular Perception for visualization and computation: the Proxima library

Supporting Information

Federico Lazzari[1], Andrea Salvadori[1], Giordano Mancini *[1], and Vincenzo Barone[1]

[1] *Scuola Normale Superiore. Piazza dei Cavalieri, 7 - 56126 Pisa*

Source Code

The current alpha version is available for evaluation purposes at *https://bitbucket.org/sns-smartlab/proxima/src/master/* .

Proxima I/O

PDB Parser

The Protein Data Bank (PDB) file format, that is the file format historically used for storing data by the PDB database of structures [1], is currently supported in Proxima. It is widely employed and almost all of the cheminformatics libraries and molecular viewers are able to parse it. Although the Protein Data Bank was recently upgradated to the mmCIF file format, its adoption is still limited; on the other hand, PDB is perhaps the most widespread molecular structure format and its use in Proxima is motivated by the huge amount of quality data available. To offer an increased access to data stored in PDB files we strived to parse and compute information from PDB file sections not always supported, such as alternate locations.

Alternate Locations and Models

In the PDB file format, each atom is allowed to have more than one location in the same model (e.g. atoms that are too movable, rotamers, symmetric molecules etc.). These different locations are called "alternate locations" in PDB terminology. Proxima stores these alternative positions in different frames of the same model. Each frame contains the same set and number of atoms, but with different properties (such as position, charge, etc.). Another property of the PDB file format is the ability to store different models within the same file (e. g. all the models compatible with a set of NMR spectra, simulation frames, etc.). In the case of a PDB file containing multiple models of the same system, alternate locations are ignored and each Proxima frame will store a different set of coordinates of the system.

Residues and Fragments

There are three special classes of residues that are explicitly treated by Proxima: i) Amino acid Residues, ii) Nucleotide Residues and iii) Generic Residue (that is a residue that does not belong to the previous classes). Proxima looks at the names of the atoms that are part of the residue, and checks whether they are compatible with the names of the atoms of one of the residues listed above (e.g. the alpha carbon atom of the amino acid residue of a peptide is typically called CA). For whatever concerns chains, instead, the implementation choice is to use Fragments instead of chains. The motivation is that the concept of "chain" is

*giordano.mancini@sns.it

something deeply related to biological systems and/or proteins. Fragments are more generally seen as chemically distinguishable portions of a molecular system. Being "chemically distinguishable" means one of two different things:

- These are two disconnected component of the system (there are no covalent bonds between these two portions).
- These two portions are made of different types of residues.

As an example, two not bonded chains are seen as different fragments even if they share the same chain identifier in the pdb file. Furthermore, if two or more polynucleotides and polypeptides are bonded together and they share the same chain identifier in the PDB file, these fragments are perceived by Proxima as different entities. Deep inspection of additional residue types will be considered in future developments.

Z-Matrix

Proxima is capable of creating a so-called Z-Matrix [2] file as an output for the Molecular Systems loaded by PDB or XYZ. This feature is important in making Proxima compatible with several computational chemistry software. The computation of the Z-Matrix in Proxima is performed by a Breadth First Search (BFS) along the molecule graph, while computing torsions and angles. [1].

Mol and Mol2 file formats

Proxima is also capable of creating mol and mol2 files [3] from molecular systems. This has proven usefull in interfacing Proxima with common Molecular Viewers. As an example, JMol [4] reads the mol file format but without performing an explicit bonds perception on the input system. The mol2 file is also required by the HTEQ software [5] that computes torsional parameters for biaryl systems.

Chemical Ring Perception

Here are shown some benchmarks for the rings perception algorithm performed on peptides made of increasing numbers of histidine residues. In particular, in Fig. S1 are reported the time of execution for the ring perception with and without the block partitioning scheme.

Parallelizing Ring Computation

A further optimization performed on our software has been to parallelize the code in threads. To do this, we used the OpenMP set of compiler directives which is supported by several free and commercial compilers (e. g. GCC, LLVM, VisualStudio). It is worth noting that while "critical" sections limit parallelism, a decent speedup on a few cores is enough for most applications. The times of execution are shown in Fig. S2. All of the benchmarks in this work have been performed on a machine equipped with a 64-bit Intel Core i7-6700 CPU (featuring 4 cores with a base clock frequency of 3.40GHz and Hyper-Threading technology) and 32GB of RAM memory.

Caffeine

In the following, we show some examples of Molecular Electrostatic Potential (MEP) surfaces computed by Proxima and visualized with the Caffeine software. In Fig. S4, the MEP iso-surfaces associated to a vanishing electrostatic potential value are shown for the benzene molecule. In Fig. S3, instead, the corresponding MEP iso-surface for the glycine molecule is shown. The green

[1]BFS is a common algorithm in Graph Theory to explore a graph G(V,E) with a worst-case complexity of $O(|V| + |E|)$, where $|V|$ is the number of vertices of the graph and $|E|$ the number of edges

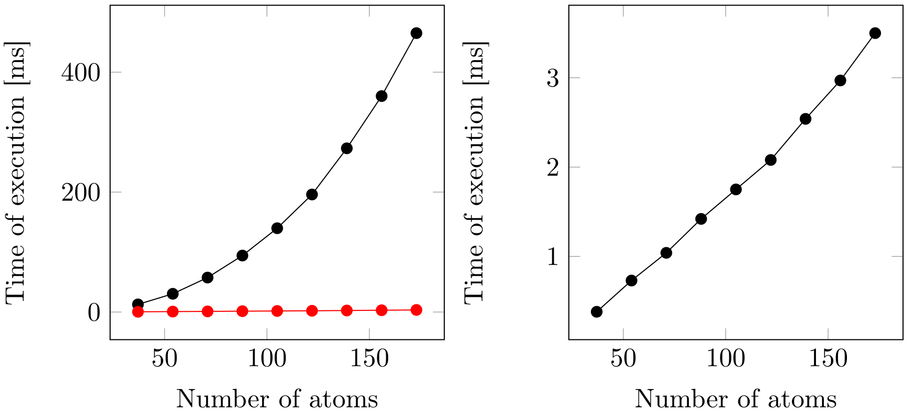

Figure S1: Times of execution on a series of histidine peptides of increasing dimensions (from 2 to ten residues). Left: Horton's algorithm without the block partitioning scheme. The red points are the same reported on the right plot. Right: Improved performance resulting from the block partitioning scheme.

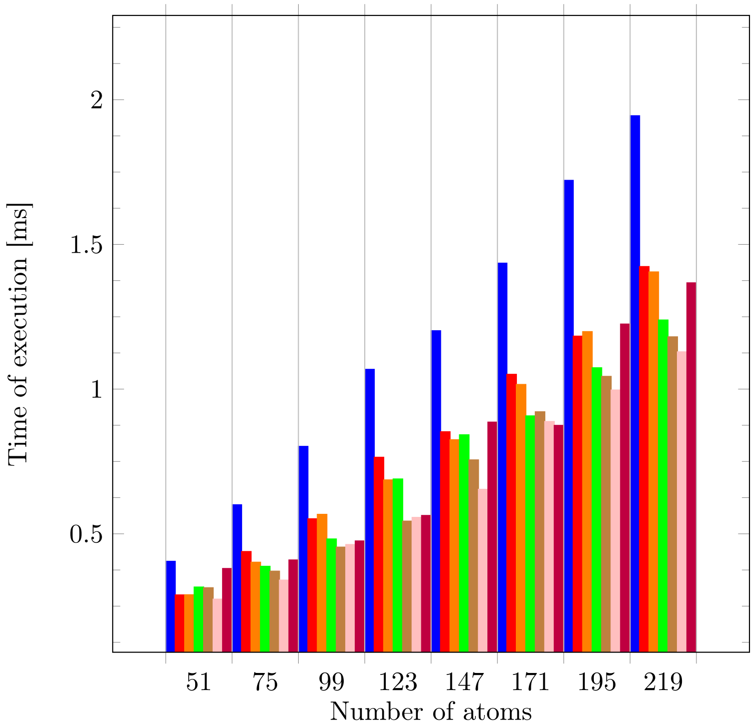

Figure S2: An histogram representing the different times of execution in relation to the number of atoms (peptides of histidine residues). Blue: 1 Thread. Red: 2 Threads. Orange: 3 Threads. Green: 4 Threads. Brown: 5 Threads. Pink: 6 Threads. Purple: 7 Threads.

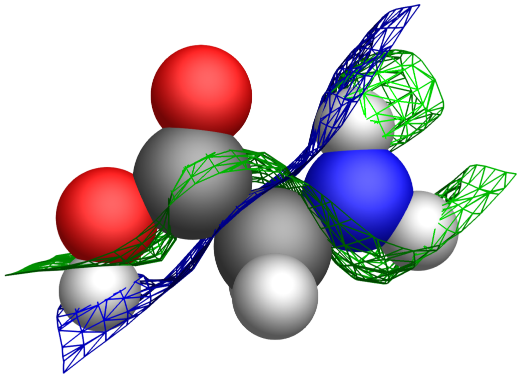

Figure S3: The MEP iso-surfaces, at 0 electrostatic potential value, for the glycine molecule. Blue surface: Computed with FQ charges. Green surface: Computed with Gasteiger charges.

surface is the one computed using the Gasteiger charges while the blue one is the one computed with the QeQ charges. It is noteworthy that the Gasteiger method tends to polarize more the individual atoms thus resulting in a more corrugated surface.

Hybridisation

The following rules are used for computing the hybridisation:

sd number of bonds < 3 AND atomic number ≥ 21 AND max angle $< 90 +$ tolerance AND min angle $>$ 90-tolerance

sp number of bonds < 3 AND (max angle + min angle)/2 $< 180 +$ tolerance AND (max angle + min angle)/2 $>$ 180-tolerance

sd^2 number of bonds < 4 AND atomic number ≥ 21 AND max angle $<$90 + tolerance AND min angle $>$ 90 - tolerance

sp^2 number of bonds < 4 AND (max angle + min angle)/2 $<$ (120+tolerance*0.55) AND (max angle + min angle)/2 $>$ (120-tolerance*0.55)

sd^3 number of bonds < 5 AND atomic number ≥ 21 AND max angle $< 109.5 +$ tolerance AND min angle $>$ 109.5 - tolerance

sp^3 number of bonds < 5 AND max angle $< 109.5 +$ tolerance AND min angle $>$ 109.5 - tolerance

sp^2d^2 number of bonds < 5 AND atomic number ≥ 21 AND max angle $< 90 +$ tolerance AND min angle $>$ 90 - tolerance

sp^3d number of bonds < 6 AND atomic number ≥ 21 AND max angle $< 180 +$ tolerance AND min angle $>$ 90 - tolerance AND there is at least a 120° angle.

sd^4 number of bonds < 7 AND atomic number ≥ 21 AND max angle $< 123 +$ tolerance AND min angle $>$ 73 - tolerance

sd^5 number of bonds < 7 AND atomic number ≥ 21 AND max angle $< 116.5 +$ tolerance AND min angle $>$ 63.5 - tolerance

sp^3d^2 number of bonds < 7 AND atomic number ≥ 21 AND max angle $< 180 +$ tolerance AND min angle $>$ 90 - tolerance

sp^3d^3 number of bonds < 8 AND atomic number ≥ 21 AND max angle $< 180 +$ tolerance AND min angle $>$ 72 - tolerance AND there is at least a 120° angle.

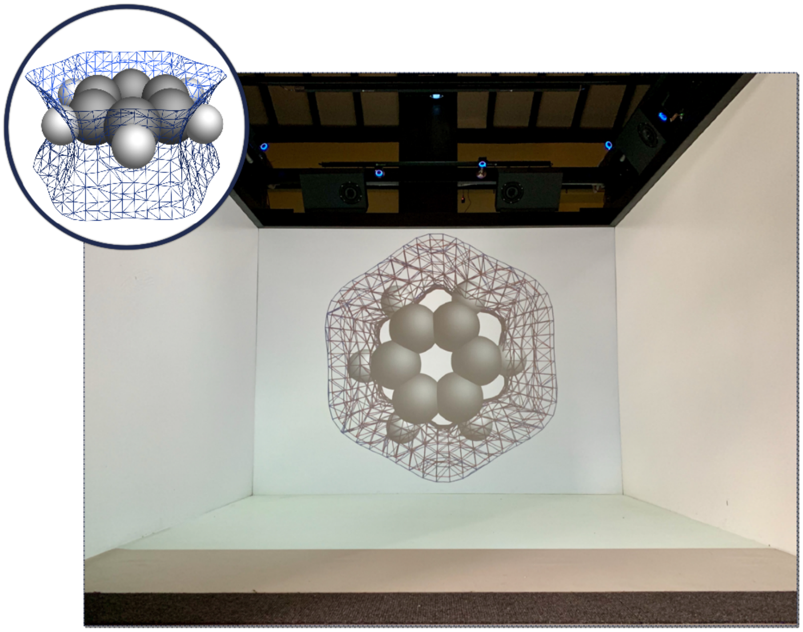

Figure S4: The MEP surface for the Benzene molecule computed with Proxima. The surface is shown in the C.A.V.E. system at the SMART laboratory of Scuola Normale Superiore [6] through the Caffeine software. [7] In the corner, the same MEP surface from a different point of view, rendered with the desktop version of the Caffeine software [7].

Here, a tolerance of 10 degrees is taken for atomic numbers below 21, and of 20 degrees otherwise. Typically, the hybridisation of terminal atoms is left unknown. There are some special cases, though, that are treated differently, such as the oxygen, the nitrogen and the carbon atoms. With regard to oxygen and nitrogen terminal atoms, the hybridisation is set to sp^2 whether they are bonded to other sp^2 atoms, sp^3 otherwise. This is reasonable since they have lone pairs and can contribute in resonance structures. With regard to the carbon terminal atoms, instead, the hybridisation of the other bonded atom is checked: If this atom has an sp^2 hybridisation, and it is not bonded to other sp^2 atoms, the hybridisation of the current terminal carbon atom is set to sp^2. Otherwise, if the other atom still has an hybridisation of sp^2 but is also bonded to other sp^2 atoms, an sp^3 hybridisation is assigned to the considered terminal atom. The same reasoning applies to the *sp* case.

PyProxima

PyProxima is a Python interface aimed at the further diffusion of the Proxima library by allowing non-C++ users to take advantage of its features. This Python module has been generated with the use of the Cython software. Most of the original methods implemented in Proxima are currently included in PyProxima and more will come in future updates. There are no external dependecies except for OpenMP (required for the parallel version of the code) and for the Eigen library (e.g. used for linear algebra calculations). To install the software library these files are required:

- The Proxima C++ source code
- The Cython source files
- The setup.py file

The installation process if fairly easy as the user has to simply type the following command in the terminal:

```
python setup.py build_ext --inplace
```

PyProxima can be used in the same way of the Proxima C++ version, with the only difference that a *Py* is required before every name. An example of this is shown below:

```
import pyproxima
pyproxima.PyAtom(1,'H',0)
```

ProximaConsole

ProximaConsole is a console application designed for using Proxima to compute bonds, hydrogen bonds, charges and molecular rings. ProximaConsole can be run with the following command:

```
ProximaConsole -i inputFile -o outputFile
```

An input file is given to the executable (pdb or xyz files are accepted as inputs) and a mol and a JSON files are generated as output. The mol file contains the geometry with the bonds computed by Proxima. The JSON file, instead, contains the additional computed rings, charges and hydrogen bonds. In order to trigger such computations additional flags can be given to ProximaConsole such as the one shown in S5.

An example of the generated JSon file is given in Fig. S6. All of the computed quantities are stored in the "*vms_proxima*" object. The "*data_mol*" object is the molecular systems, with the covalent bonds computed by Proxima, stored in the mol file format. Instead, the "*vms_charges*" array stores the charges of the atoms (in the same order of the atom indexes). The "*vms_frags*" array stores fragments such as the rings: each fragment is an object described by a name (the "*fr_name*" value)

-cycles This flag is used for computing rings with the Horton's algorithm.

-gasteiger This flag is used for computing charges with the Gasteiger method.

-fq This flag is used for computing charges with the FQ method.

-hbonds This flag is used for computing hydrogen bonds.

-mep This flag is used for computing the electrostatic potential and output it as a cube file.

-spin This flag is followed by the total electron spin for the given system.

-charge This flags is followed by the total charge of the given system.

Figure S5: ProximaConsole additional flags

and the set of indexes of the atoms that take part of the fragment (the "*fr_index*" array). Finally, the "*vms_hbonds*" array stores hydrogen bonds. Each hydrogen bond is an object described by a "*hb_force*" value that identifies the strenght of the hydrogen bond and, again, an array of indexes ("*hb_atoms*") of the atoms that take part of the hydrogen bonds in the order [donor, hydrogen, acceptor]. The ProximaConsole software is a compiled software available for Windows, Linux and macOS under request.

Complementary Tools

The PyProxima and ProximaConsole tools allow users to employ directly the library as preprocessing tool (in a way akin to e.g. Antechamber [8] or to import and use it in a Python based enviroment or pipeline. To further increase the flexibility, we devised an interactive GUI for non-expert users. This is called ProximaGUI and is based on the widespread JMol software [4] for the visualization of chemical structures. ProximaGUI is a compiled software avialable for Windows, Linux and macOS under request.

```
{
    "section_run": {
        "program_info": {
            "program_name": "Proxima"
        },
        "section_single_configuration_calculation": {
            "vms_proxima": {
                "data_mol": "\n Proxima\n\n ... M END\n",
                "vms_charges": [ −0.113679, ... , 0.0765165 ],


                "vms_frags": [
                    {
                        "fr_name": "cycle 1 − 5 bonds",
                        "fr_index": [ 8, 9, 10, 12, 13 ]
                    },
                    ...
                    {
                        "fr_name": "cycle 2 − 5 bonds",
                        "fr_index": [ 28, 29, 30, 32, 33 ]
                    }
                ],
                "vms_hbonds": [
                    {
                        "hb_force": 0.491506,
                        "hb_atoms": [ 21, 4, 36 ]
                    },
                    ...
                    {
                        "hb_force": 0.62624,
                        "hb_atoms": [ 24, 1, 16 ]
                    }
                ]
            }
        }
    }
}
```


Figure S6: An example JSON file

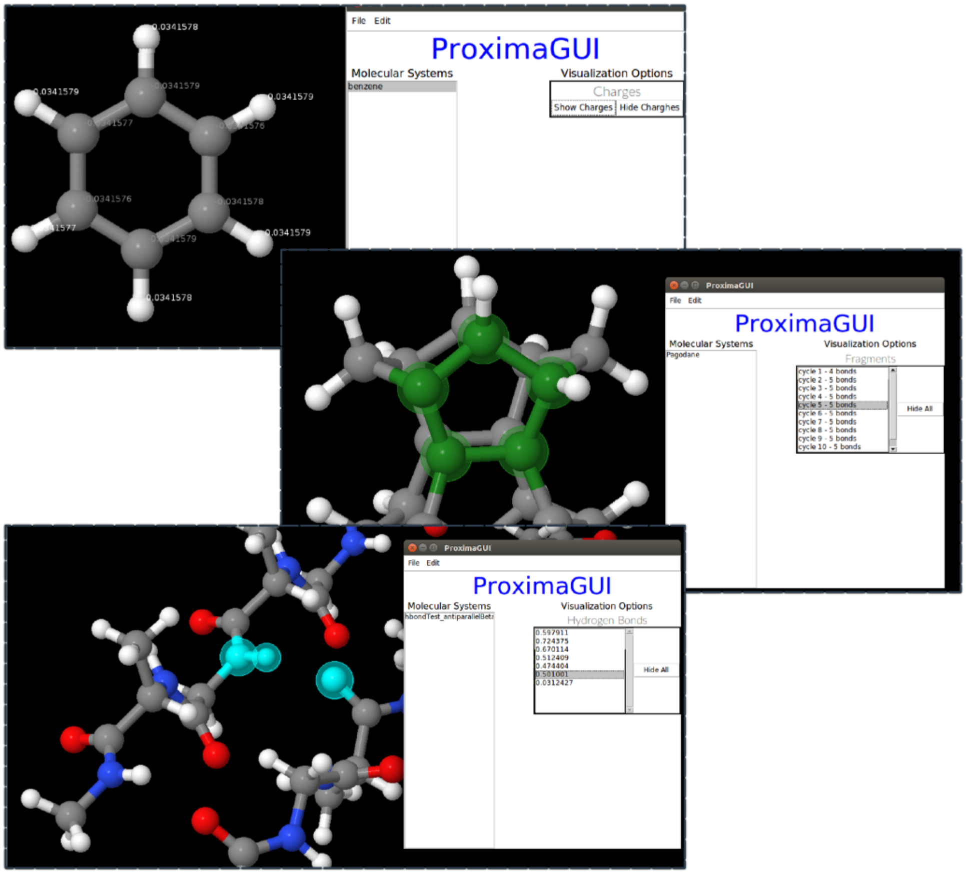

Figure S7: Representative screenshots showing charges, rings and hydrogen bonds computed with ProximaGUI

References

[1] Helen Berman, Kim Henrick, and Haruki Nakamura. Announcing the worldwide protein data bank. *Nat. Struct. Biol.*, 10:980 EP, Dec 2003. Correspondence.

[2] Constructing z-matrices. https://gaussian.com/zmat/. Accessed: 2019-11-04.

[3] Mol file format. https://goldbook.iupac.org/terms/view/MT06966. Accessed: 2019-11-04.

[4] Jmol: an open-source browser-based html5 viewer and stand-alone java viewer for chemical structures in 3d. http://jmol.sourceforge.net/. Accessed: 2019-06-04.

[5] Zhaomin Liu, Stephen J. Barigye, Moeed Shahamat, Paul Labute, and Nicolas Moitessier. Atom types independent molecular mechanics method for predicting the conformational energy of small molecules. *J. Chem. Inf. Model.*, 58(1):194–205, Jan 2018.

[6] Smart sns - theoretical and computational chemistry at sns. http://smart.sns.it. Accessed: 2019-05-05.

[7] Andrea Salvadori, Gianluca Del Frate, Marco Pagliai, Giordano Mancini, and Vincenzo Barone. Immersive virtual reality in computational chemistry: Applications to the analysis of qm and mm data. *Int. J. Quantum Chem.*, 116(22):1731–1746.

[8] Romelia Salomon-Ferrer, David A. Case, and Ross C. Walker. An overview of the Amber biomolecular simulation package. *WIREs Comput. Mol. Sci.*, 3(2):198–210, March 2013.
